# Supplementary material for: Microbiome Migration from Soil to Leaves in Maize and Rice
Source: Microorganisms. 2025 Apr 20;13(4):947. doi: 10.3390/microorganisms13040947 (PMC12029745; doi:10.3390/microorganisms13040947)
Supplement: Supplementary file 1 [file microorganisms-13-00947-s001.zip › Supplementary Tabie S1 and Figure.pdf]

Table S1. Relationships Between 22 Bacterial Genera and Plant Functions

Genus of strain	Growth promotion	Abiotic Stress Resistance	Biotic Stress Resistance	References
Bacillus	+	+	+	[51,100-102]
Achromobacter	+	+	+	[51,94-96,98]
Escherichia-Shigella	+			[103]
Pantoea	+	+	+	[104-106]
Serratia	+			[107,108]
Chromobacterium	+		+	[109,110]
Acinetobacter	+		+	[111-113]
Pseudomonas	+	+	+	[114-116]
Bradyrhizobium	+			[117,118]
Sphingobium	+	+		[119,120]
Xanthomonas	-	-	-	[121,122]

Note: The "Genus of strain" refers to the genera of the 22 strains. A "+" indicates a positive effect on the corresponding plant function, "-" indicates an inhibitory effect on the plant, and a blank space indicates that no related effects have been found so far.

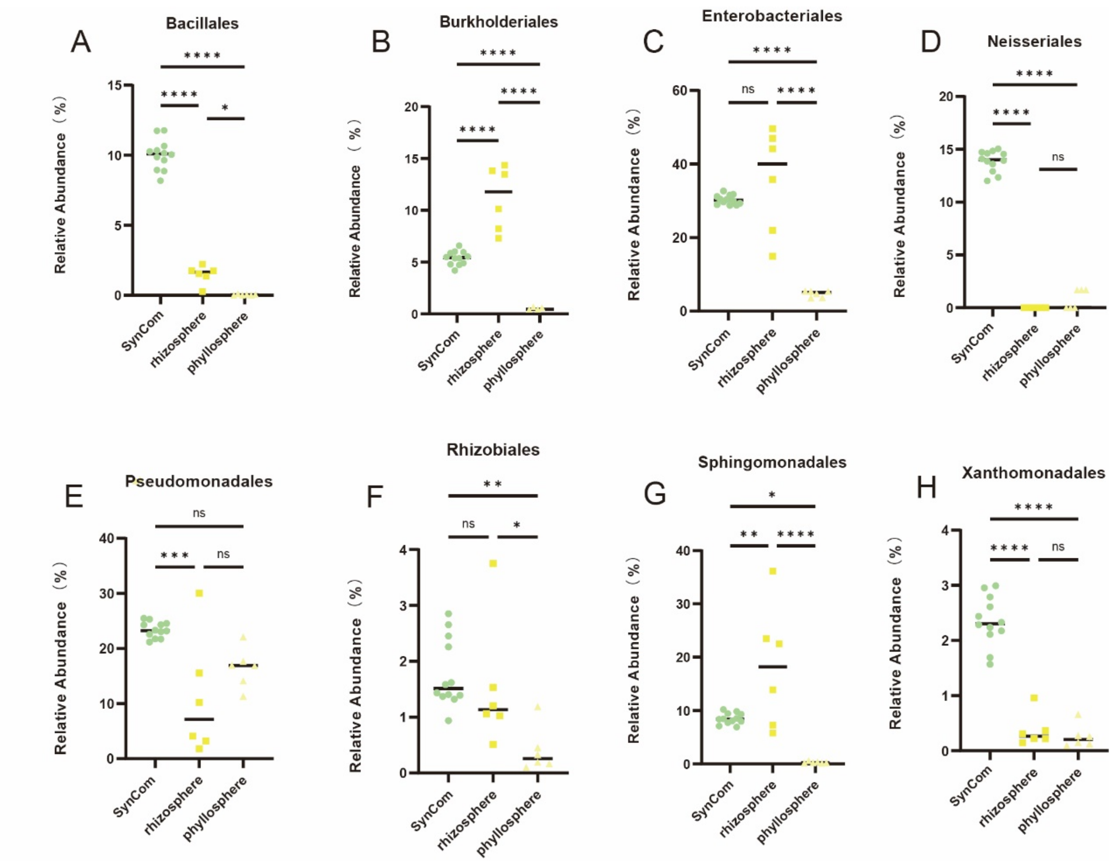

Supplementary Figure S1. Presents a variance analysis of the abundance values of SynCom, rice rhizosphere, and rice phyllosphere microbiota at the order level. The orders analyzed include: **(A)** Bacillales; **(B)** Burkholderiales; **(C)** Enterobacteriales; **(D)** Neisseriales; **(E)** Pseudomonadales; **(F)** Rhizobiales; **(G)** Sphingomonadales; **(H)** Xanthomonadales. Symbols of different colors represent different plant compartments, with green, bright yellow, and light yellow representing SynCom, rhizosphere, and phyllosphere, respectively. With significance levels indicated as follows: ns ($p > 0.05$), * ($p \leq 0.05$), ** ($p \leq 0.01$), *** ($p \leq 0.001$). and **** ($p < 0.0001$).

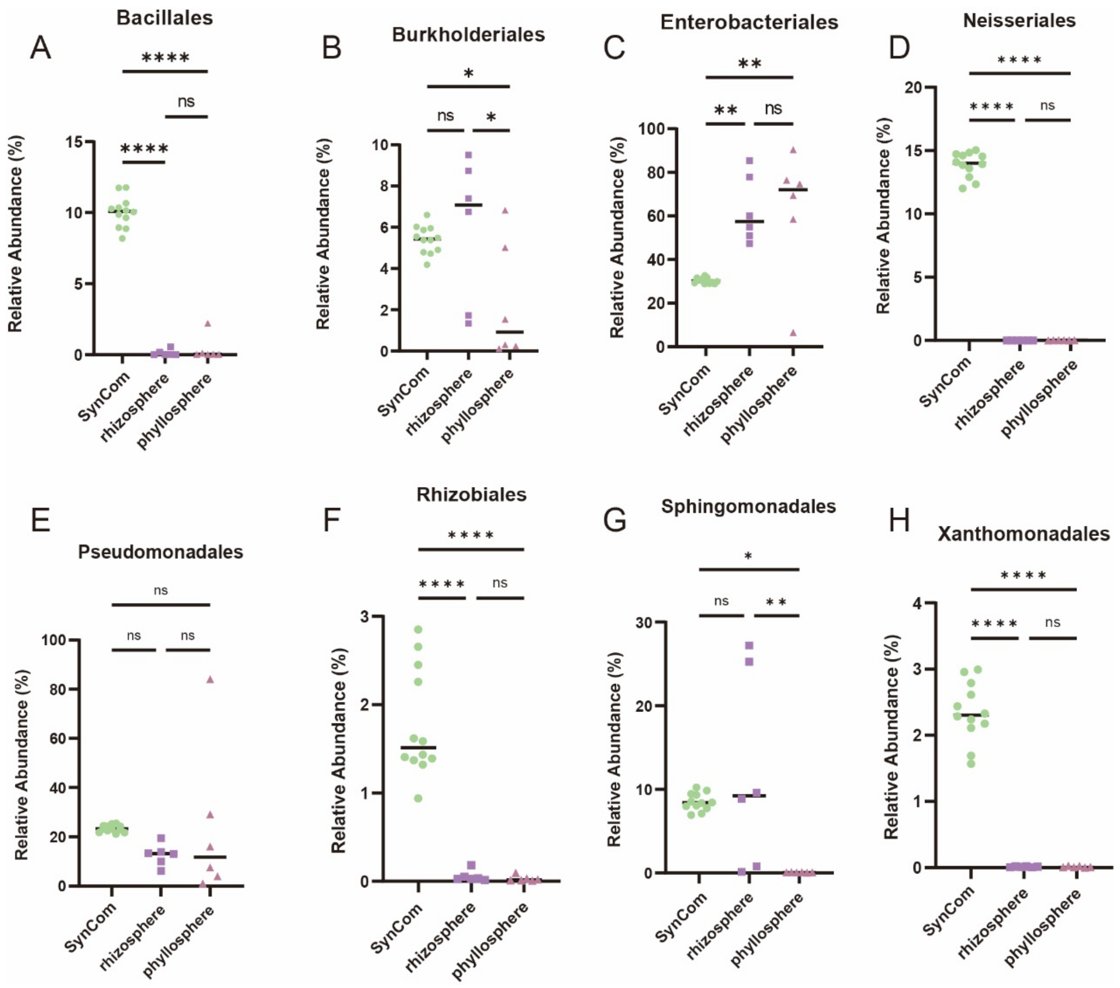

Supplementary Figure S2. Presents a variance analysis of the abundance values of SynCom, maize rhizosphere, and maize phyllosphere microbiota at the order level. The orders analyzed include: (A) Bacillales; (B) Burkholderiales; (C) Enterobacteriales (D) Neisseriales; (E) Pseudomonadales; (F) Rhizobiales; (G) Sphingomonadales; (H) Xanthomonadales. Symbols of different colors represent different plant compartments, with green, purple and pink representing SynCom, rhizosphere, and phyllosphere, respectively. With significance levels indicated as follows: ns ($p > 0.05$), * ($p \leq 0.05$), ** ($p \leq 0.01$), *** ($p \leq 0.001$). and **** ($p < 0.0001$).

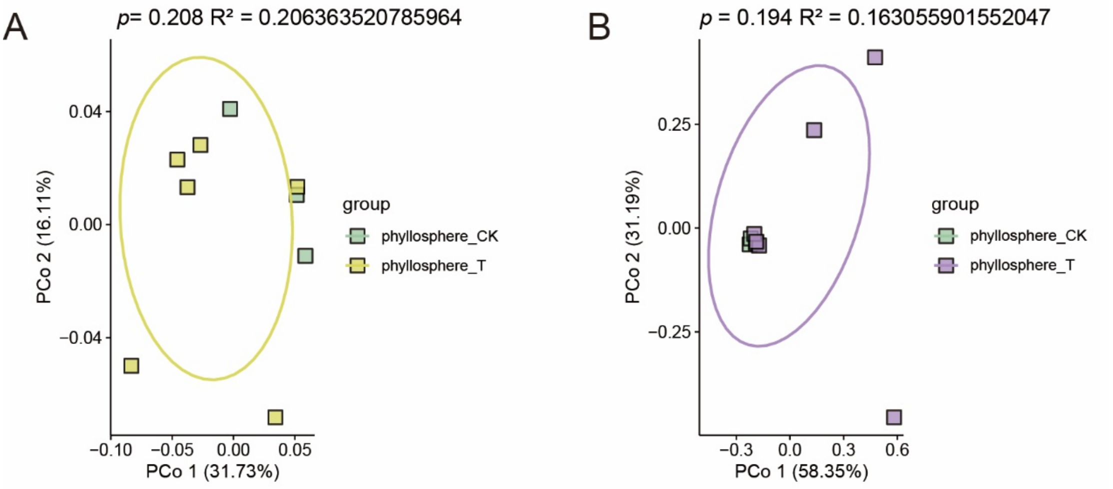

Supplementary Figure S3. Unconstrained PCOA of bacterial communities in the phyllosphere of maize and rice inoculated and uninoculated with SynCom (A) Shows an unconstrained PCOA based on Bray-Curtis distance for the principal coordinates PCO1 and PCO2, illustrating the clustering of bacterial communities in the phyllosphere of rice, inoculated and uninoculated with SynCom *(p*-value calculated using one-way Permanova). Ellipses cover 68% of the data for each group. **(B)** Shows an unconstrained PCOA based on Bray-Curtis distance for the principal coordinates PCO1 and PCO2, illustrating the clustering of bacterial communities in the phyllosphere of maize inoculated and uninoculated with SynCom (*p*-value calculated using one-way Permanova). Ellipses cover 68% of the data for each group.
